# Intestinal Barrier Function in Chronic Kidney Disease

**DOI:** 10.3390/toxins10070298

**Published:** 2018-07-19

**Authors:** Björn Meijers, Ricard Farré, Sander Dejongh, Maria Vicario, Pieter Evenepoel

**Affiliations:** 1 Department of Microbiology and Immunology, KU Leuven, 3000 Leuven, Belgium; sander.dejongh@student.kuleuven.be (S.D.); pieter.evenepoel@uzleuven.be (P.E.); 2 Division of Nephrology, UZ Leuven, 3000 Leuven, Belgium; 3 Translational Research Center for Gastrointestinal Disorders (TARGID), KU Leuven, 3000 Leuven, Belgium; ricard.farre@kuleuven.be; 4 Centro de Investigación Biomédica en Red de Enfermedades Hepáticas y Digestivas (CIBERehd), Instituto de Salud Carlos III, 28029 Madrid, Spain; maria.vicario@vhir.org; 5 Laboratory of Translational Mucosal Immunology, Digestive Diseases Research Unit, Vall d’Hebron Institut de Recerca, 08035 Barcelona, Spain

**Keywords:** uremia, CKD, intestinal barrier, inflammation

## Abstract

The kidneys are key contributors to body homeostasis, by virtue of controlled excretion of excessive fluid, electrolytes, and toxic waste products. The syndrome of uremia equals the altered physiology due to irreversible loss of kidney function that is left uncorrected for, despite therapeutic intervention(s). The intestines and its microbial content are prime contributors to this syndrome. The intestinal barrier separates the self (or the so-called “milieu intérior”) from the environment. In the large intestine, the intestinal barrier keeps apart human physiology and the microbiota. The enterocytes and the extracellular mucin layer functions form a complex multilayered structure, facilitating complex bidirectional metabolic and immunological crosstalk. The current review focuses on the intestinal barrier in chronic kidney disease (CKD). Loss of kidney function results in structural and functional alterations of the intestinal barrier, contribution to the syndrome of uremia.

## 1. Introduction

The kidneys are key contributors to body homeostasis, by virtue of controlled excretion of excessive fluid, electrolytes, and toxic waste products. The effects of alterations in kidney function on body homeostasis are complex. Our basic understanding is that a reduced kidney function will lead to less excretion of inorganic and organic solutes, thus leading to altered body homeostasis [1]. However, particularly longer-term alterations in kidney function will lead to much more profound changes in body homeostasis.

Chronic kidney disease is defined as structural and/or functional abnormalities of the kidneys that are present for at least three months [2]. For most patients it denotes the progressive loss of kidney function, ultimately leading to end-stage kidney disease (ESKD). This condition often is referred to as uremia. In essence, uremia is a so-called remnant syndrome [1], as it does not represent the natural history of chronic kidney disease. Instead, it equals the altered physiology due to irreversible loss of kidney function despite therapeutic intervention(s).

One important characteristics of chronic kidney disease is retention of organic solutes. Often, these are referred to as uremic retention solutes (URS). Metabolomics studies point to retention of numerous as yet unidentified solutes [3]. The relative contribution of the microbial metabolism towards the mammalian internal environment has long been underestimated. Modern metabolomics techniques now allow to fully appreciate this contribution [4]. It has become clear that the microbial metabolism complements mammalian host physiology. Numerous studies now point to the gut as a prime contributor to the uremic internal environment [3,5,6,7,8].

A second key feature of chronic kidney disease is the high incidence of inflammation and malnutrition [9,10,11]. In the Chronic Renal Insufficiency Cohort (CRIC), plasma levels of the cytokines interleukin1β (Il-1β), the interleukin-1 receptor antagonist (IL-1RA), interleukin-6 (IL-6), tumor necrosis factor (TNF)-α, as well as high sensitivity C-reactive protein (CRP) and fibrinogen were higher among participants with reduced levels of estimated glomerular filtration rate (eGFR) [12], and are associated with progressive loss of kidney function [13]. Only recently, the role of the gut in CKD associated inflammation has been recognized [14,15].

As more and more data point to a key role of the gut in the syndrome of uremia, it is worthwhile to have a closer look at the gut, and especially the intestinal barrier, which is the frontier between the organism and the outside world.

## 2. The Host-Microbiota Interaction in Health and Disease

The human gut, and especially the large intestine, is colonized by trillions of microorganisms. These microbiota encode at least 150-fold more genes than the human genome, together referred to as the microbiome [16]. Humans may thus be considered “supraorganisms” or “holobionts”. Dominant members of the gut microbiome are similar across the globe and are independent of race and gender [17]. However, community structure and abundance profiles vary significantly among geographically separated human populations, suggesting that environmental selection at the local scale (especially diet) determines the gut microbiome. This plasticity constitutes a mechanism by which the holobiont can rapidly adapt to environmental changes that require capabilities beyond what is encoded by the human genome [17].

For millions of years, the gut microbiome has co-evolved with the host. The cohabitation provides microorganisms with a stable environment, while microorganisms provide the host with a broad range of functions such as digestion of complex dietary macronutrients, defense against pathogens, production of nutrients and vitamins, maintenance of the immune system. This biological symbiosis is now jeopardized by unprecedented changes in human lifestyle and environment. Antibiotic use and other drug therapy, diets poor in dietary fiber and rich in red meat, excessive sanitation and birth by Caesarean section are all known to have profound effects on the gut microbiota [18].

Recent data also indicate that chronic kidney disease associates with a distinct gut microbiota composition [19,20,21] and cause microbial metabolism to shift towards a predominantly proteolytic fermentation pattern [8,22,23,24]. Lacking prospective cohort studies, however, it is hard to define whether changes in gut microbiota and metabolism are cause or consequence of chronic kidney disease.

Similar to the skin, the intestinal barrier is the physiological barrier that separates the self (or the so-called “milieu intérior”) from the environment. It is important to acknowledge that the colonic intestinal wall not only acts as a gatekeeper protecting against the invasion of pathogens and toxins but also as a choirmaster of the immune system-microbiota alliance. When operating optimally, this alliance interweaves the innate and adaptive arms of immunity in a dialog that selects, calibrates, and terminates responses in the most appropriate way [25].

A better insight in complex crosstalk between the host and the gut microbiota in health and disease may open new avenues for (individualized) therapy. The present review focuses on the structure and functionalities of the colonic intestinal barrier both in health and in CKD.

## 3. Physiology of the Intestinal Barrier

The anatomy and physiology of the intestinal barrier varies across the length of the intestinal tract. A broad division can be made between the small intestine, which has a lower exposure to bacteria and usually is not colonized, and the large intestine, which is home to the largest and most diverse microbial community residing within the human body. In this review, we will focus mostly on the intestinal barrier of the large intestine, although some data also suggest CKD-related alterations in the intestinal barrier of the upper gastro-intestinal tract [26].

The gastro-intestinal tract is lined by a single epithelial cell layer. It consists predominantly of enterocytes, interspersed with specialized cell-types such as goblet cells and neuro-endocrine cells. Direct contact between the epithelial cell layer and the gut microbiota is limited by a specialized mucus layer, produced by goblet cells and enterocytes [27]. The mucus composition also differs across the length of the gastro-intestinal tract, with the small intestine having a single mucus layer, whereas the stomach and large intestine have a bilayer, i.e., the inner and outer mucous layer [27]. These two layers differ in composition and function. The outer layer provides the habitat for the commensal flora, whereas the inner mucous layer is impermeable to bacteria, functioning as an antimicrobial shield to protect the enterocytes from direct contact with bacteria.

The inner mucous layer is produced by the goblet cells and consists predominantly of the gel-forming MUC2 mucin that is cross-linked by six different covalent and noncovalent bonds [28]. Under certain circumstances, the inner mucous layer becomes permeable, allowing bacteria to reach the epithelial cells in large quantities. This is a common mechanism that underlies many forms of acute or chronic colonic inflammation [29].

The outer mucous layer equally contains MUC2 mucin. The ultrastructural characteristics however are altered by host protease activity, increasing average pore size facilitating penetrance into the mucous layer. The mucin-bound carbohydrate layer provides energy for the adherent bacteria, together with the complex carbohydrates from dietary sources that escaped digestion in the small intestine.

The mucous bilayer, predominantly secreted by the goblet cells, is the first line of defense against microbiota. The enterocytes itself also produce a number of membrane-bound mucins. These mucins consist of a large highly glycosylated extracellular mucin domain as well as a cytoplasmic domain in interaction with the cytoskeleton. Some of these cytoplasmic tails may regulate the externalization of several ion channels such as the sodium/hydrogen exchanger 3 (NHE3) and the cystic fibrosis transmembrane conductance regulator (CFTR) [30].

Behind the barrier formed by the complex multilayered mucins, the enterocytes form a second line of defense between the gut lumen and mammalian milieu interne. The enterocyte plasma membrane prevents transcellular flux of most hydrophilic solutes. Transcellular transport almost exclusively consists of actively regulated transport. To prevent paracellular flux of luminal solutes, the intestinal epithelial cells are sealed together by the zonula occludens complex (tight junction).

Despite the fact that the intestinal barrier is composed of a multilayered structure, thus shielding the human internal environment from the colonic microbial content, there is intense cross-talk between both compartments. Broadly speaking, this bidirectional communication consists of both metabolic as well as immunological signals, aiming to maximize the win-win between commensal microbiome and the host.

## 4. Metabolic Pathways Bridging the Intestinal Barrier

Tight junctions are selectively permeable and capable of discriminating between solutes on the basis of either size and/or charge. At least two distinct routes are involved in paracellular permeability [31]: a charge selective pathway for ions, small uncharged solutes and water, called the pore pathway, and a charge-independent pathway for medium-sized (up to 10–20 KDa) molecules called the leak pathway.

**Figure 1:**
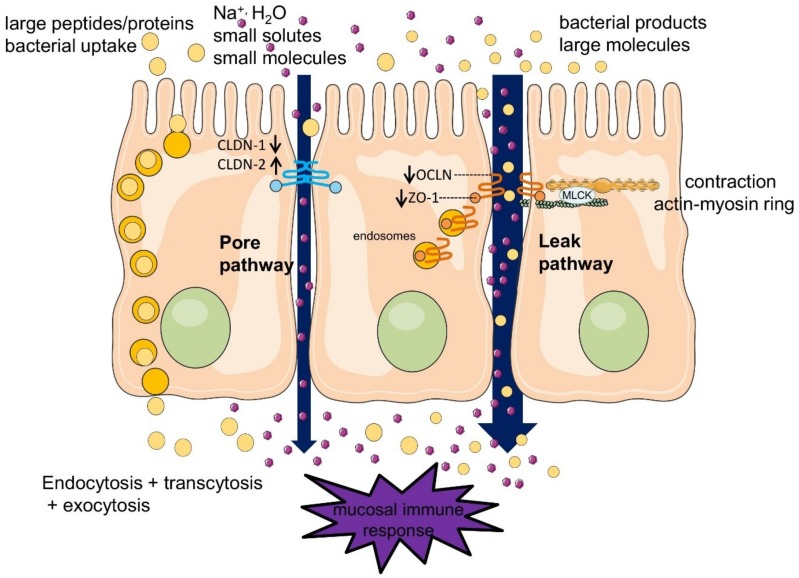
Paracellular and transcellular transport in the intestinal epithelium. The enterocytes form a polarized single cell layer. The apical side, characterized with villi, is in contact with the intestinal lumen. The epithelial cells are tied together by the tight junction complexes. Several transporter pathways facilitate transport from the apical to the basolateral side. Transcellular transport is mediated by active transporters or by endocytosis–transcytosis–exocytosis. Paracellular transport is via either the pore pathway or the leak pathway.

The pore pathway is mainly determined by the claudin (CLDN) family, of which the best studied proteins are CLDN1 and CLDN2 [32,33,34]. The pathophysiological consequences of increased permeability of the pore pathway are unclear, since it is clear that immunogenic peptides or bacterial products cannot pass through it. The leak pathway is regulated by the myosin II light chain (MLC) phosphorylation by the MLC kinase (MLCK) that is accompanied by contraction of the apical peri-junctional actomyosin ring of enterocyte resulting in opening of the paracellular space [35,36]. The contraction of this ring is a fast way of regulating the barrier function in contrast to the slower regulation of the pore pathway that involves gene transcription. The leak pathway is mainly regulated by ZO-1 and occludin (OCLN), facilitating passage of medium-sized hydrophilic molecules up to 10–20 kDa. Larger peptides, proteins, and whole bacteria or large bacterial products are endocytosed into vesicles and transported through the cells via transcytosis and basolateral exocytosis [37] (Figure 1). It is important to emphasize that a large proportion of bacteria translocated to the mesenteric lymph node and eventually to the portal circulation cross the epithelial barrier via M-cells located at the Peyer’s patches.

Nutrients as glucose, amino acids, peptides, vitamins but also bile acids present in the intestinal lumen cannot cross the intestinal epithelium by the transcellular or paracellular pathways and they enter the enterocyte and then reach the portal circulation via different membrane transporters. Moreover, uptake and efflux transporters allow the movement of molecules in the opposite direction to keep blood levels of potentially toxic compounds low. Interestingly, transporters are one of the determinants of the pharmacokinetics of high number of orally administered drugs. While much research has been focused on the role of transporters in the liver and kidney, less is known about the importance and the types of transporters in the intestine [38].

Membrane drug transporters are classified into the solute carrier (SLC) and the ATP-binding cassette (ABC) transporter super-families, both include more than 400 transporters [39,40]. Apical ABC efflux transporters are highly abundant in enterocytes and function as pumps that extrude toxins and drugs out of the enterocyte, thereby limiting the intestinal absorption of many clinically important and frequently orally prescribed drugs such as statins, antibiotics, immunosuppressive drugs and drugs commonly used to prevent or treat cardiovascular diseases.

## 5. Immunological Cross-Talk

Gut homeostasis is maintained by the strategical location of the gut associated lymphoid tissue (GALT) at the mucosal interface, allowing surveillance and innate and adaptive effector functions of immunocytes. Intestinal epithelial cells cooperate in this immune defense, as they express receptors, process and present antigens, and release mediators that facilitate bidirectional communication with the immune system. Innate Immunity is the first line of immunological defense against potentially luminal harmful substances. The main cellular elements of the innate response are enterocytes, goblet and Paneth cells, as well as subepithelial neutrophils, dendritic cells, macrophages, eosinophils, and mast cells. Innate responses are triggered by pathogen-recognition receptors (PRRs), which serve as sensors of pathogen-associated molecular patterns (PAMPs) present in pathogenic and commensal bacteria. PRRs activate inflammatory responses characterized by nuclear factor-κB (NF-κB) activation; subsequent chemokine and cytokine production, recruitment and immune cells activation; and release of antimicrobial molecules (defensins and cathelicidins) [37].

Adaptive immunity, essential for the development of tolerance, is triggered by antigen exposure and is characterized by immunological memory which confers long-term protection to the host.

Often, the metabolic and immunological signaling are discussed as two separate dedicated pathways. Several lines of evidence point to closely intertwined systems, where metabolites interfere with immunological signaling and vice versa.

## 6. Intestinal Barrier and CKD

Vaziri et al. were the first to explore the intestinal permeability and its structural components in animals, using experimentally 5/6th nephrectomy induced CKD. They observed intense infiltrates of lymphocytes into the lamina propria [41]. Using the same experimental model of CKD, they equally demonstrated a profound effect of experimentally induced CKD on the structural components of the intestinal barrier in the upper gastro-intestinal tract [26], as well as in the large intestine [41,42].

These experiments predicted augmentation of intestinal permeability in CKD, as a result of the loss of tight junction proteins. Animal experiments confirmed the loss of intestinal integrity and increased translocation of living bacteria across the intestinal barrier into the liver, in combination with increased serum levels of bacterial endotoxin [20].

It has been hypothesized that increased gut permeability contributes to inflammation in patients with advanced CKD [43,44,45]. Indeed, some studies report increased levels of circulating endotoxin, i.e., the biologically active LPS complex associated with the outer membrane of gram-negative bacteria, in patients with CKD. Other studies however could not confirm a relationship between levels of endotoxin and stages of CKD [46,47]. Given the short half-life and the limitations of the limulus amebocyte lysate (LAL) assay, measurement of endotoxin might not be the best assay to study exposure to bacterial fragments. We studied concentrations of soluble CD14 (sCD14) as marker of host response to exposure to endotoxin. Concentrations of sCD14 increase parallel to the loss of kidney function and are associated with overall and cardiovascular mortality [47]. Although these data demonstrate augmented exposure to endotoxins, they cannot be seen as definite proof of increased permeability of the intestinal barrier in CKD. Additional serum markers of increased intestinal permeability have been looked at, predominantly serum levels of bacterial DNA. These data further corrobated the concept of increased permeability of the intestinal barrier to bacterial fragments in patients with advanced CKD [48]. However, the exact mechanism how bacterial DNA reaches the systemic circulation is unknown.

## 7. Intestinal Transporters and CKD

The number of metabolites that cross the intestinal barrier via active transport is near-endless, ranging from nutrients, bacterial metabolites as well as pharmaceutical agents. Surprisingly little is known about the role of these transporters in patients with advanced CKD. For some metabolites, more data are available. These will be discussed, as an illustration of the complex alterations in the regulation of active transport across the intestinal barrier in CKD.

Intestinal excretion accounts for approximately one-third of total body clearance of uric acid (UA), whereas kidney clearance accounts for the other two-thirds. In physiological conditions, kidney clearance is mainly regulated by the apical SLC2A9 together with the basolateral SLC22A6 (OAT1) and SLC22A8 (OAT3) transporters, whereas the apical *ABCG2* transporter is the key intestinal transporter. The basolateral transporter responsible for the uptake of UA from the blood into the enterocyte is unknown. SLC22A6 and SLC22A7 are potential candidates since they are expressed in the basolateral side of the enterocytes and are able to transport UA [38]. The available data suggest that the intestinal uric acid handling mainly depends on the apical *ABCG2* [49], as *ABCG2* knockout mice have higher concentrations and lower intestinal excretion of uric acid [50] and the *ABCG2* variant Q141K (rs2231142) was found to be associated with elevated serum concentrations of uric acid [50]. Interestingly, research in CKD animal models show that the upregulation of *ABCG2* in the intestine triggered by an elevation of serum UA may play a compensatory role in its increasing intestinal excretion [51,52].

Several other URS are associated with cardiovascular complications and poor survival in chronic kidney disease [53,54,55]. Clinical studies demonstrate that serum concentrations of these solutes are determined both by their renal clearance and by the intestinal adsorption [56]. Whether the systemic accumulation of URS, including p-cresol sulfate (PCS) and indoxyl sulfate (IS) of gut origin, involves intestinal uptake and efflux transporters needs further investigations. Results in rat renal cortical slices show that the organic anion transporter (OAT1/OAT3) play an important role in the uptake of IS and PCS in the kidney [57,58,59,60]. In the intestine, preliminary data indicate a role for the multidrug resistance-associated protein (MRP) family (personal communication R. Poesen).

Recently, Shinozaki et al. provided evidence for another altered intestinal transporter system, mediated by the organic cation transporter 1 (OCTN1)-ergothinoneine axis [61]. The carnitine/organic cation transporter 1 (OCTN1) is located at the apical side of enterocytes. It is the key transporter for food-derived ergothioneine, a naturally occurring anti-oxidant found in foods such as grains, mushrooms and beans [62]. Silencing the *SLC22A4* gene, encoding OCTN1, in cultured cells inhibited uptake, and *SLC22A4* knockout mice exhibited complete deficiency of ergothioneine. These findings suggest that OCTN1 is essential for intestinal absorption of ergothioneine, and that no escape mechanism is available [63]. In a mouse model of CKD, intestinal absorption of ergothioneine was clearly diminished [61]. This could be attributed to a disturbed OCTN1 localization on the intestinal apical cellular membrane. Proteomic analysis, RT-PCR, western blotting, and immunohistochemistry revealed that PDZ (PSD95, Dlg, and ZO1), a PDZK1 domain-containing protein that regulates the localization of transporters, was decreased in mice with CKD [61].

In CKD patients, the dysregulation of drug transporters [49] has to be considered since the pharmacokinetics of patients’ medication can be potentially altered. Indeed, results from preclinical models and clinical investigations have demonstrated that drug transporters-mediated non-renal clearance are dramatically impaired in CKD [64]. Accumulated uremic toxins may not only downregulate but also directly inhibit the activity of drug transporters.

## 8. Conclusions

**Figure 2:**
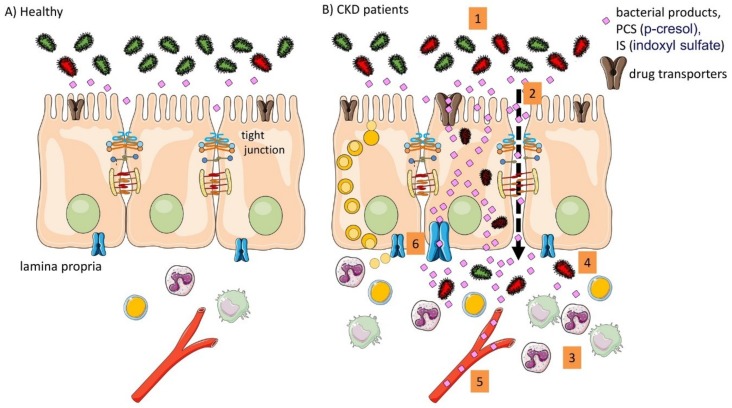
Summary of the intestinal implication in the pathophysiology in CKD. (**A**) In healthy conditions the intestinal barrier function is intact; (**B**) In CKD different alteration were described in pre-clinical animal models and in patients. (1) altered microbiota composition (dysbiosis); (2) impaired barrier function; (3) intestinal immune activation; (4) bacterial translocation; (5) systemic inflammation and presence of bacterial products; (6) altered drug transporters.

The intestinal barrier separates the self (or the so-called “milieu intérior”) from the environment, shielding mammalian physiology from the outside world. In the large intestine, the intestinal barrier shields the eukaryotic milieu interne from direct contact with the microbiota. Its complex multi-layered structure, consisting of the enterocytes and the extracellular mucin layer functions as a tight seal. At the same time, the composition of the mucin, the characteristics of the tight junctions and a host of active transporter systems allow for a complex bidirectional metabolic and immunological crosstalk. The loss of kidney function interferes with the functions of the intestinal barrier. As a result, the effectiveness of the intestinal barrier is reduced and exposure to bacterial products is increased. CKD also interacts in complex ways with the active transport mechanisms, thus altering body homeostasis of numerous solutes. Our understanding of the effects of CKD on these processes is far from complete (Figure 2).
